# Treatment of Habitual Patellar Dislocation in an Adult by Isolated Medial Patellofemoral Ligament Reconstruction

**DOI:** 10.1155/2014/647272

**Published:** 2014-03-04

**Authors:** Yoann Bohu, Mathieu Thaunat, Nicolas Lefevre, Shahnaz Klouche, Serge Herman, Yves Catonné

**Affiliations:** ^1^Groupe Hospitalier Universitaire La Pitié Salpêtrière, AP-HP, 75013 Paris, France; ^2^Institut de l'Appareil Locomoteur Nollet, 75017 Paris, France; ^3^Clinique du Sport Paris V, 75005 Paris, France

## Abstract

Habitual patellar dislocations are rare in adults. Treatment is difficult, and often associated with significant morbidity. A 30-year-old man, construction worker, presented a habitual patellofemoral dislocation which was caused by direct trauma to the knee as a child. Clinical examination showed a 3 cm leg-length discrepancy with no rotational deformities. The patient had a limp and loss of function; the patella was dislocated laterally and had locked at 20° of flexion with a range of motion of 0°/0°/30°. Open surgery was performed associating lateral retinacular release, reconstruction of the medial patellofemoral ligament with an ipsilateral gracilis tendon graft. The postoperative course was simple with no complications. Four months after surgery the patient has begun working normally. At the final 50-month clinical follow-up, knee range of motion was 0°/0°/130°, and functional results were excellent on clinical assessment scores of Kujala, Lysholm, and subjective IKDC. MPFL reconstruction alone seems effective in habitual posttraumatic patellar dislocation in adults without any associated bone anomalies.

## 1. Introduction

The origin of recurrent patellar instability is usually posttraumatic. Dislocation results in irreversible injuries due to lateral translation of the patella [1, 2]. Hawkins et al. [3] reported 40% rates of patellofemoral pain and 70% rates of instability after dislocation. Several factors predisposing to patellar instability have been described such as trochlear dysplasia or a patella alta [4]. Recently several studies have shown that the medial patellofemoral ligament (MPFL) reconstruction in the treatment of patellar instability in adults had a high success rate [5] but with a complication rate of 26% [6]. Few cases of habitual dislocations have been reported [7].

## 2. Case Presentation

**Figure 1:**
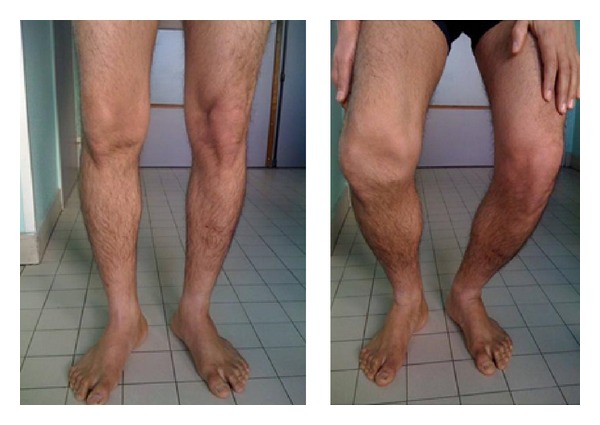
Preoperative assessment: the patella is in place in extension and dislocated in flexion.

A 30-year-old man consulted for limping. In the past three years, the patient had been having trouble walking, could not squat down, or go up and down stairs. The patient's history included a knee injury at the age of 5 in a road accident. He did remember neither precise diagnosis nor functional or surgical previous treatment. There were no scars on the knee. The patient was 1.76 m tall and weighed 68 kg with varus knee morphology and no leg-length discrepancy. Placing weight on one foot resulted in lateral patellar dislocation, and squatting was impossible. The patella was mobile, but systematically dislocated laterally at 30° of flexion (Figure 1).

**Figure 2:**
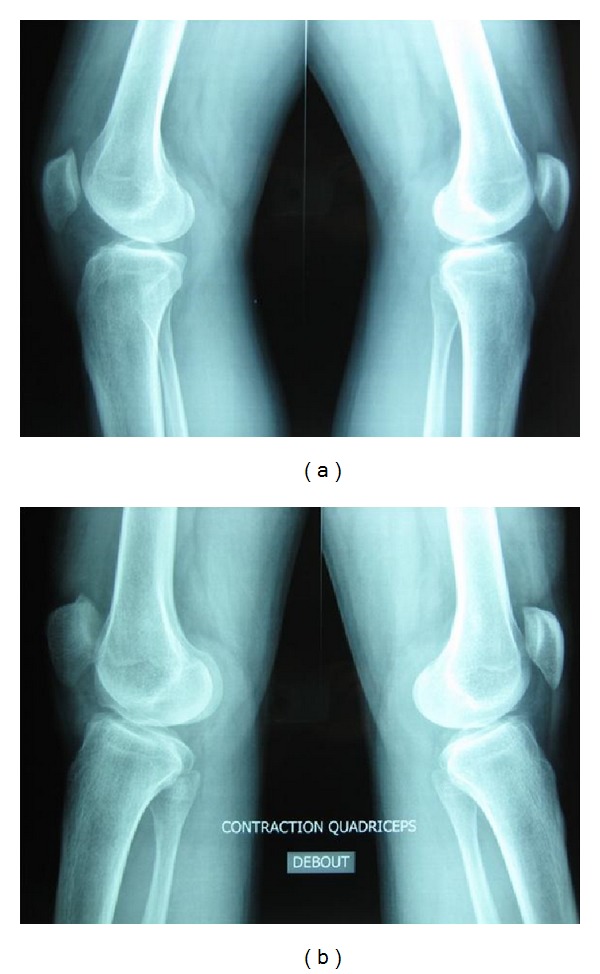
Comparative weight-bearing X-ray lateral view: (a) without contraction of the quadriceps and (b) with contraction of the quadriceps.

**Figure 3:**
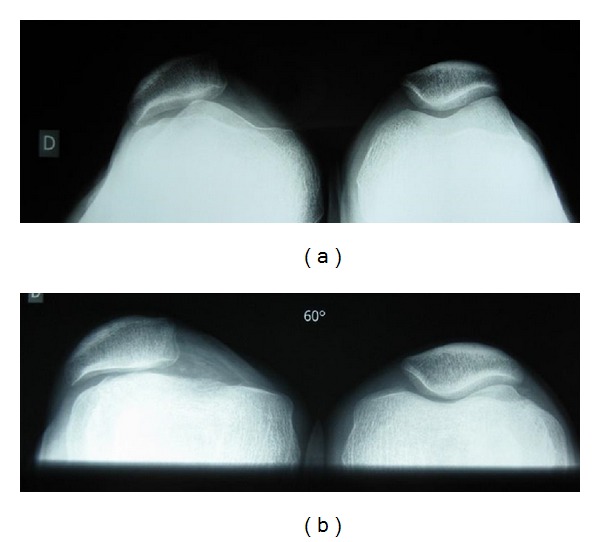
Preoperative patellofemoral X-ray, tangential view in neutral rotation: (a) 30° flexion and (b) 60° flexion.

The rest of the clinical examination, in particular the neurological, muscular, and tendon results were normal. X-ray confirmed genu varum, showing a patellar tilt angle of 30°. The trochlear morphology (groove and depth) was normal as well as the height of the patella with Caton-Deschamps index equal to 1 [8]. There was no patellar dysplasia, but the trochlea was slightly flattened (Figures 2 and 3).

The medial border of the patella was calcified. There was no associated leg dysplasia. CT scans of leg rotation showed physiological tibial torsion and femoral anteversion. Femoral anteversion measured on CT scan between the femoral neck angle and the biepicondylar axis was symmetrical, 12° on the right and 13° on the left. The preoperative Kujala et al. score [9] was 41%. The preoperative Lysholm score was 6%. The subjective IKDC score was 10% for pain, 17% for symptoms, and 17% for recreational and sports activities, or a global IKDC of 14.7%. The diagnosis of habitual posttraumatic patellofemoral dislocation was made. The indication for reconstruction of the stabilizing ligament of the patella was based on the significant functional incapacitation and the lack of appropriate conservative treatment.

An open surgery procedure was performed. First lateral retinacular release of the patella was performed. Then the ipsilateral gracilis tendon was harvested at its distal insertion with a tendon stripper. The graft was 12 cm long. Two holes were drilled into the patellar bone by a medial parapatellar approach. After pulling a suture through the holes the femoral attachment point was found and with the help of a temporary pin in the medial epicondyle, favorable anisometry could be obtained (the graft relaxed as the knee was flexed) and a Corkscrew suture anchor (Arthrex, Naples, FL, USA) was put in place. After confirming patellar centering during 4 to 6 flexion-extension cycles, the suture anchor was secured in its final position. The graft was placed into the drill holes then secured to the suture anchor and doubled back on itself as described by Thaunat and Erasmus [10, 11]. The vastus medialis (vastus internus) was then placed below and outside the patella and secured with “U-” stitches to provide overlapping reinforcement. The postoperative course was simple with a brace to walk for 45 days and immediate rehabilitation at between 0 and 80° of flexion.

**Figure 4:**
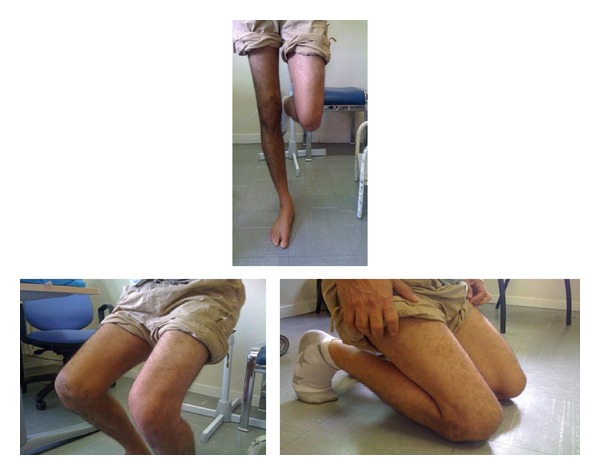
Postoperative photographs: stability, no dislocation.

**Figure 5:**
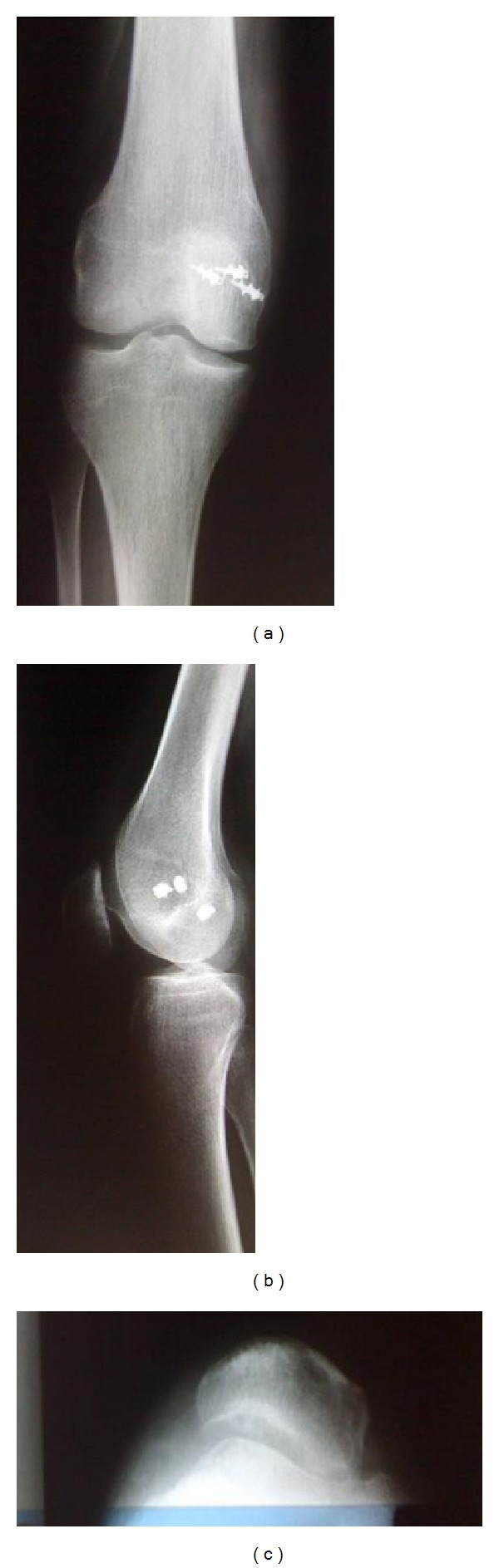
Postoperative X-rays of the knee: (a) anteroposterior, (b) lateral, and (c) tangential patellofemoral views at 60°.

At 4 months of follow-up, the patient was able to work normally as construction worker in public works. At the final 50-month clinical follow-up, the operated knee was stable and there was no pain. There was no recurrent dislocation or apprehension. There was full range of motion (Figures 4 and 5).

Postoperative X-rays show the patella recentered at 60°. The patient was very satisfied with the results.

Results of knee function were excellent on functional scales for patellar instability with significant improvement of all clinical scores. The Kujala score was 83/100 points and the Lysholm score 90/100. The global IKDC score was 90.8%. The items for pain, symptoms, and leisure and sports activities were scored 93.3%, 90%, and 90%, respectively.

## 3. Discussion

To our knowledge, no other cases of habitual patellofemoral dislocation in young adults secondary to trauma which occurred in childhood have been described in the literature.

According to the Andrish clinical score for children [12], this patient had “habitual” rather than “fixed” traumatic dislocation. This instability was secondary to a childhood injury suggested by the patient's history and the features of medial patellar calcifications found on X-ray, whereas 90% of patellofemoral instability cases in children and adolescents are nontraumatic [13]. The initial medial femoropatellar injuries never healed. Imaging of the soft tissues might have provided more specific information. An MRI or ultrasound might have shown features of the MPFL and the attachment of the vastus medialis (internus) on the patella, but these results were not available for the preoperative work-up.

No predisposing factor was found [4]. Morphological assessment of the patellofemoral compartment did not reveal any signs of dysplasia. On the femoral side, there was no crossing sign or spurs, and the depth of the trochlear groove was normal. Patella tilt was increased in a lateral view during contraction of the quadriceps but the morphology of the patella was normal. The height of the patella was normal with no recurvatum.

The injuries had not affected skeletal development during growth. There was no leg-length discrepancy. Femoral anteversion on CT scan was identical to the contralateral side. The quadriceps was not retracted. Overall, the cause of dislocation was isolated rupture of the medial stabilizing apparatus of the patella.

Because the injury was anatomical, conservative treatment with rehabilitation was not proposed. The first line therapeutic strategy was surgical.

Numerous techniques have been described in the literature for the treatment of patellar dislocations. These included either lateral retinacular release or proximal or distal realignment. Aglietti et al. [14] and Sherman et al. [15] have reported a failure rate for lateral retinacular release of 44% and 25%, respectively. As already mentioned by Fithian et al. [16], we believe that the primary frontal stabilizer of the patella is the MPFL.

Numerous studies have had good midterm results (at least 1-year follow-up) with MPFL reconstruction. In a study of 14 patients Drez et al. [17] reported 93% good and excellent results at 39 months of follow-up in 15 patients with a mean age of 26 years. Howells et al. [5] found no recurrence of dislocation at a mean 16-month follow-up in 193 patients with a mean age of 22 years and a statistically significant functional improvement for all patients reviewed.

In our patient, radiographic results have shown that instability was not caused by bone anomalies. We believed that the MPFL was torn, making normal patellar tracking impossible. Therefore, there was no indication for recentering the patella or lowering the tibial tubercle by osteotomy. Isolated repair of MPFL was possible because there was no retraction of the knee extensor apparatus or predisposing bone factors, which would have required further surgical procedures.

The main difficulty which could have been encountered during surgery was identifying a flexion deficit after performing reconstruction because of the secondary retraction of the quadriceps. By increasing tension in the graft to reduce the patella during flexion, we could obtain stiffness after 90°. During surgery we adjusted graft tension with a pin in the medial epicondyle attachment. We adjusted the length of the graft by taking into account engagement of the patella and by obtaining full range of motion in the knee. The graft was tense close to extension and relaxed during flexion.

Criteria which allowed him to return to work were no pain, stability of the patella, no swelling, and full range of motion. We chose to assess treatment using functional scores. Although the Lysholm, Kujala, and IKDC scores have been validated to evaluate this disease, the score by Kujala et al. [9] is more specific and sensitive for patellofemoral damage. The improvement from 41 to 83% in the latter score was perfectly correlated to the functional improvement of the patient, who was able to begin working as a construction worker (kneeling, squatting, climbing, lifting, carrying, etc.).

## 4. Conclusion

In case of no retraction of the knee extensor apparatus or predisposing bone factors, isolated MPFL reconstruction seems effective in treating confirmed patellar instability. In case of stiffness or associated bone anomalies (trochlear dysplasia, patella alta) other therapeutic procedures would be necessary. This case was original because of the habitual dislocation and of the excellent functional results obtained after simple surgery and a short postoperative follow-up. This report shows that a case of neglected traumatic patellofemoral dislocation in childhood can progress to habitual patellofemoral dislocation in young adult.
